# Pulmonary rehabilitation improves exercise capacity, health-related quality of life, and cardiopulmonary function in patients with non-small cell lung cancer

**DOI:** 10.1186/s12885-024-11977-5

**Published:** 2024-02-15

**Authors:** Chun-Yao Huang, Min-Shiau Hsieh, Po-Chun Hsieh, Yao-Kuang Wu, Mei-Chen Yang, Shiang-Yu Huang, I-Shiang Tzeng, Chou-Chin Lan

**Affiliations:** 1 Division of Pulmonary Medicine, Department of Internal Medicine, Taipei Tzu Chi Hospital, Buddhist Tzu Chi Medical Foundation, 289 Jianguo Road, Xindian District, New Taipei City, 23142 Taiwan; 2 School of Medicine, Tzu Chi University, Hualien, Taiwan; 3 Division of Thoracic Surgery, Taipei Tzu Chi Hospital, Buddhist Tzu Chi Medical Foundation, New Taipei City, Taiwan; 4 Department of Chinese Medicine, Taipei Tzu Chi Hospital, Buddhist Tzu Chi Medical Foundation, New Taipei City, Taiwan; 5 School of Post-Baccalaureate Chinese Medicine, Tzu Chi University, Hualien, Taiwan; 6 Division of Respiratory Therapy, Taipei Tzu Chi Hospital, Buddhist Tzu Chi Medical Foundation, New Taipei City, Taiwan; 7 Department of Research, Taipei Tzu Chi Hospital, Buddhist Tzu Chi Medical Foundation, New Taipei City, Taiwan

**Keywords:** Exercise capacity, Health-related quality of life, Lung cancer, Pulmonary rehabilitation

## Abstract

**Background:**

Lung cancer significantly impairs exercise capacity and health-related quality of life (HRQL). Pulmonary rehabilitation (PR) has demonstrated positive effects on exercise capacity and HRQL in lung cancer patients. However, its impact on cardiopulmonary function needs further exploration. The aim of this study was to explore the effects of PR on cardiopulmonary function, exercise capacity and HRQL in patients with lung cancer.

**Methods:**

Patients with lung cancer were enrolled in a 12-week PR program. Each participant underwent a thorough evaluation, which included spirometry, cardiopulmonary exercise testing, respiratory muscle strength test, and evaluation of HRQL using the Chronic Obstructive Pulmonary Disease Assessment Test (CAT).

**Results:**

Fifty-six patients completed the PR program. Following PR, exercise capacity significantly improved, as evidenced by increased peak oxygen uptake and work rate (both *p* < 0.05). Exertional symptoms were notably reduced, including leg soreness and dyspnea at peak exercise, accompanied by a decrease in the CAT score (all *p* < 0.05). Furthermore, improvements in cardiopulmonary function were observed, encompassing respiratory muscle strength, ventilatory equivalent, tidal volume, stroke volume index, and cardiac index at peak exercise (all *p* < 0.05).

**Conclusions:**

PR demonstrated notable enhancements in cardiopulmonary function, exertional symptoms, exercise capacity, and HRQL in patients with lung cancer.

## Background

Lung cancer is a devastating disease that poses a significant global health burden [1]. It is one of the most prevalent and deadliest cancers, accounting for a substantial number of cancer-related deaths worldwide [1]. With its high incidence and mortality rates, lung cancer not only affects physical health but also has a profound effect on overall well-being and health-related quality of life (HRQL) [2]. According to recent statistics, lung cancer is the leading cause of cancer-related deaths worldwide [2, 3]. The World Health Organization estimated that approximately 2.21 million new cases of lung cancer were diagnosed in 2020, with 1.8 million deaths [2, 3]. Hence, there is an urgent need for effective interventions to address the burden of lung cancer and improve patient outcomes.

The seriousness of lung cancer stems not only from its high mortality rate, but also from its debilitating symptoms [2]. Patients often experience respiratory distress, coughing, chest pain, fatigue, and a decline in physical function, which significantly impact exercise capacity, HRQL, and cardiopulmonary function [4]. Low exercise capacity can affect treatment outcomes in patients with lung cancer [5]. It increases the risk of surgical complications, hinders the effectiveness of chemotherapy, exacerbates radiation-induced lung injury, and negatively impacts the tolerability of further therapy [5].

Low exercise capacity is often accompanied by a decline in overall physical function and HRQL [5]. This contributes to a sedentary lifestyle, further exacerbating the negative impact on health and increasing the risk of complications. Low exercise capacity is associated with mortality in patients with lung cancer [6]. Reduced exercise capacity is indicative of compromised cardiopulmonary function. Patients with reduced exercise capacity may have limited ability to tolerate chemotherapy or radiotherapy, thereby affecting treatment efficacy, and potentially compromising survival [6].

It is crucial to develop comprehensive treatment strategies that go beyond conventional cancer therapy. Pulmonary rehabilitation (PR) are defined by the European Respiratory Society and American Thoracic Society (ATS/ERS) as “a comprehensive intervention based on a thorough patient assessment followed by patient-tailored therapies that include, but are not limited to, exercise training, education, and behavior change, designed to improve the physical and psychological condition of people with chronic respiratory disease and to promote the long-term adherence to health-enhancing behaviors” [7, 8]. It is a potential intervention for patients with lung cancer. PR improves overall exercise capacity and HRQL in patients with lung cancer [9]. However, little is known about the effects of PR on cardiopulmonary function in patients with lung cancer. Therefore, the aim of this study was to investigate the effects of PR on exercise capacity, HRQL, and cardiopulmonary function in patients with lung cancer.

## Materials and methods

### Study design and patient recruitment

Patients diagnosed with lung cancer were recruited from the outpatient department to participate in the PR program. The inclusion criteria were patients with stable disease who underwent a cardiopulmonary exercise test (CPET). "Stable disease" indicates that there are no significant changes in tumor size or metastasis, indicating that the cancer is not progressing. Patients who were unable to complete the CPET due to orthopedic or neurological impairments were excluded. The study protocol was approved by the Ethics Committee of Taipei Tzu Chi Hospital (IRB no: 12-X-088). Informed consent was obtained from all participants. All patients underwent spirometry, CPET, respiratory muscle strength testing, symptom evaluation at peak exercise, and HRQL assessment using the Chronic Obstructive Pulmonary Disease Assessment Test (CAT).

### Pulmonary function

Pulmonary function was measured using a spirometer (Medical Graphics Corp., St. Paul, MN, USA). The measurements were performed in accordance with the American Thoracic Society guidelines [10].

### CPET

CPET was performed using a bicycle ergometer (Lode Corival, Groningen, The Netherlands) and an incremental protocol. Breeze Suite 6.1 (Medical Graphics Corp.) was utilized for breath analysis, which included the following variables: oxygen uptake (VO2), carbon dioxide output (VCO2), tidal volume (VT), and respiratory frequency (RF). Heart rate (HR), blood pressure (BP), and arterial oxygen saturation (SpO2) were monitored simultaneously [11]. The anaerobic threshold (AT) was determined using the V-slope method [11]. Work efficiency (WE) was determined by linear regression analysis of the ratio of VO2 to work rate (WR) [12]. Oxygen pulse (O2P) was determined by dividing VO2 by HR [13]. The ventilatory equivalent (VEQ) was calculated as the ratio of VCO2 to minute ventilation (VE) at nadir during CPET [14].

### Respiratory muscle strength

The maximum inspiratory pressure (MIP) and maximum expiratory pressure (MEP) were measured using a respiratory pressure meter (Micro Medical Corp., England). For the MIP measurements, patients were instructed to exhale until reaching the residual volume, followed by a rapid and forceful maximal inspiration. For the MEP measurements, patients were instructed to inhale until reaching the total lung capacity, and then exhale with maximal effort [12].

### Cardiac performance

Physioflow (Manatec Biomedical, Poissy, France), a noninvasive hemodynamic monitoring device, was used to measure the stroke volume index (SVI) and cardiac index (CI) during CPET. Physioflow uses thoracic impedance cardiography to assess changes in blood flow and cardiac parameters [15]. Electrodes were placed on the thorax. The device measures changes in impedance caused by pulsatile blood flow. Based on these impedance changes, Physioflow calculated the SVI and CI.

### Dyspnea and leg fatigue score at peak exercise

Dyspnea and leg fatigue at peak exercise during CPET were evaluated using the Borg scale (a 10-point scoring system for evaluating symptom severity). A higher score on the Borg scale indicates more severe symptoms [16].

### HRQL

The Taiwan Society of Pulmonary and Critical Care Medicine has made the Chinese version of the CAT available at http://tspccm.org.tw. The CAT comprises eight items that assess various symptoms of chronic obstructive pulmonary disease, including cough, phlegm, chest tightness, breathlessness, limited activities, confidence in leaving home, sleeplessness, and energy [4]. Each item was scored from 0 to 5, resulting in a total CAT score ranging from 0 to 40. A higher score indicates more severe symptoms. A total CAT score of ≥ 10 indicates a high symptom burden [4]. In the previous study, the CAT significantly reflected the HRQL in patients with lung cancer [4].

### PR program

In the 12-week hospital-based PR program, all patients attended two sessions per week. Each session of PR encompassed exercise training, breathing exercises including pursed-lip and diaphragmatic breathing, education on medication, self-management techniques, and airway clearance strategies. The exercise training was conducted using a cycle ergometer. The training program in each section involved a progression of intensities starting at 50% of peak VO2 for the first 10 min, increasing to 60% of peak VO2 for the next 10 min, and finally 80% of peak VO2 for the last 20 min. The peak VO2 was guided based on the data obtained from the pre-PR CPET. Each session had a duration of 40 min and was supervised by a respiratory therapist. Vital signs, including SpO2, RF, HR, and BP, were monitored and recorded.

Out of the 70 patients who were arranged for pre-PR CPET to assess exercise capacity and cardiopulmonary function, 14 did not complete PR and did not undergo post-PR CPET. The remaining 56 patients completed PR, and their post-PR CPET results were included in the analysis. The dropout rate in this study was 20%.

### Statistical analysis

All parameters are expressed as mean ± SD. Statistical analyses were conducted using SPSS version 24.0 (SPSS Inc., Chicago, IL, USA). A paired *t*-test was used to compare parameters before and after PR. The threshold for statistical significance was set at *p* < 0.05.

## Results

### Baseline demographic characteristics

The baseline characteristics of the 56 patients are shown in Table 1. The mean age was 63.2 ± 10.5 years (body weight, 59.5 ± 9.1 kg; body height, 158.7 ± 8.1 cm). Seventeen patients (30.4%) were male; 39 (69.6%) were female. One patient (1.8%) was a current smoker, 10 (17.9%) were ex-smokers, and 45 (80.3%) were non-smokers. The smoking history among the participants was 6.7 ± 16.4 pack-years. Thirty-seven patients (66.1%) had stage I disease, seven (12.5%) had stage II disease, six (10.7%) had stage III disease, and six (10.7%) had stage IV disease.

**Table 1:** Baseline characteristics

Characteristics	Mean ± SD or n (%)
Age (years)	63.2 ± 10.5
Body weight (kg)	59.5 ± 9.1
Body height (cm)	158.7 ± 8.1
Sex	
Male	17 (30.4%)
Female	39 (69.6%)
Smoking status	
Current smoker	1 (1.8%)
Ex-smoker	10 (17.9%)
Non-smoker	45 (80.3%)
Smoking amount (pack-years)	6.7 ± 16.4
Tumor stage	
I	37 (66.1%)
II	7 (12.5%)
III	6 (10.7%)
IV	6 (10.7%)
Cancer type	
Adenocarcinoma	50 (89.3%)
Squamous cell carcinoma	6 (10.7%)
Treatment	
Surgery	49 (87.5%)
Chemotherapy	9 (16.1%)
Radiotherapy	6 (10.7%)
COPD	7 (12.5%)
Asthma	1 (1.8%)
ECOG-PS	
0	6 (10.7%)
1	43 (76.8%)
2	7 (12.5%)

*Abbreviation*: *SD* Standard deviation, *n* Number of patients, *COPD* Chronic obstructive pulmonary disease, *ECOG-PS* Eastern Cooperative Oncology Group performance status

### Effects of PR on exercise capacity, symptoms during exercise, and HRQL

The pre- and post-PR outcomes of exercise capacity, symptoms during exercise, and HRQL are shown in Table 2. The WR improved significantly from 78.8 ± 28.3 to 84.5 ± 25.9 W (mean difference: 5.7 ± 16.3 W; *p* = 0.011), and from 92.1 ± 28.3% to 103.4 ± 26.4% (mean difference: 11.3 ± 22.2%; *p* < 0.001). The peak VO2 increased from 1,069.6 ± 245.2 to 1,141.1 ± 278.7 mL/min (mean difference: 71.5 ± 182.7 mL/min; *p* = 0.005), and from 79.0 ± 16.8% to 83.4 ± 15.7% (mean difference: 4.5 ± 11.9%; *p* = 0.007). Regarding symptoms during exercise, leg soreness and exertional dyspnea decreased from 4.1 ± 1.6 to 3.6 ± 1.4 (mean difference: –0.5 ± 1.6; *p* = 0.029) and from 4.6 ± 2.1 to 3.7 ± 1.7 (mean difference: –0.9 ± 1.9; *p* = 0.001), respectively. The total CAT score decreased from 11.4 ± 5.6 to 9.5 ± 4.4 (mean difference: –1.9 ± 4.7; *p* = 0.004). Improvements were observed in phlegm (from 1.4 ± 1.1 to 1.1 ± 0.9 [mean difference: − 0.3 ± 1.0; *p* = 0.042]), breathlessness (from 1.8 ± 1.2 to 1.5 ± 1.0 [mean difference: –0.3 ± 1.1; *p* = 0.046]), and sleeplessness (from 1.5 ± 1.2 to 1.3 ± 1.1 [mean difference: –0.2 ± 0.8; *p* = 0.041]) scores, but not in coughing (*p* = 0.312), chest tightness (*p* = 0.249), limited activities (*p* = 0.118), confidence in leaving home (*p* = 0.055), and energy (*p* = 0.109) scores.

**Table 2:** Effects of PR on exercise capacity, symptoms at peak exercise, and HRQL

	**Pre-PR**	**Post-PR**	**Mean difference**	***p***
Maximal WR (watt)	78.8 ± 28.3	84.5 ± 25.9	5.7 ± 16.3	0.011
Maximal WR (%)	92.1 ± 28.3	103.4 ± 26.4	11.3 ± 22.2	< 0.001
Peak VO2 (mL/min)	1,069.6 ± 245.2	1,141.1 ± 278.7	71.5 ± 182.7	0.005
Peak VO2 (%)	79.0 ± 16.8	83.4 ± 15.7	4.5 ± 11.9	0.007
Leg soreness during exercise	4.1 ± 1.6	3.6 ± 1.4	− 0.5 ± 1.6	0.029
Dyspnea during exercise	4.6 ± 2.1	3.7 ± 1.7	− 0.9 ± 1.9	0.001
Total CAT score	11.4 ± 5.6	9.5 ± 4.4	− 1.9 ± 4.7	0.004
Cough	1.8 ± 1.0	1.7 ± 0.9	− 0.1 ± 0.9	0.312
Phlegm	1.4 ± 1.1	1.1 ± 0.9	− 0.3 ± 1.0	0.042
Chest tightness	1.7 ± 1.2	1.6 ± 1.1	− 0.2 ± 1.1	0.249
Breathlessness	1.8 ± 1.2	1.5 ± 1.0	− 0.3 ± 1.1	0.046
Limited activities	0.8 ± 0.8	0.6 ± 0.8	− 0.2 ± 0.8	0.118
Confidence in leaving home	0.9 ± 1.2	0.6 ± 1.0	− 0.3 ± 1.2	0.055
Sleeplessness	1.5 ± 1.2	1.3 ± 1.1	− 0.2 ± 0.8	0.041
Lack of energy	1.4 ± 1.1	1.2 ± 1.0	− 0.2 ± 0.9	0.109

*p* = comparison between pre- and post-PR

*Abbreviations*: *CAT* Chronic Obstructive Pulmonary Disease Assessment Test, *HRQL* Health-related quality of life, *PR* Pulmonary rehabilitation, *VO2* Oxygen uptake, *WR* Work rate

### Effects of PR on respiratory parameters

The pre- and post-PR outcomes of the respiratory parameters are shown in Table 3. No significant differences were observed between pre- and post-PR measurements of forced expiratory volume in 1 s (FEV1)/ forced vital capacity (FVC) %, FEV1, and FVC (all *p* > 0.05). MIP improved significantly from 69.5 ± 24.3 to 75.1 ± 24.2 cmH2O (mean difference: 5.6 ± 17.4 cmH2O; *p* = 0.019). No significant improvement was observed in MEP (*p* = 0.870). During exercise, VT increased significantly from 1,129.8 ± 277.2 to 1,211.3 ± 357.4 mL (mean difference: 81.5 ± 265.9 mL; *p* = 0.026). RF did not differ at rest or during exercise (*p* = 0.489 and 0.490, respectively). SpO2 also did not differ at rest or during exercise (both *p* > 0.05). VEQ decreased from 36.5 ± 5.1 to 35.1 ± 4.6 (mean difference: –1.3 ± 4.9; *p* = 0.048).

**Table 3:** Effect of PR on respiratory responses to exercise

	**Pre-PR**	**Post-PR**	**Mean difference**	***p***
FEV1/FVC (%)	81.0 ± 8.6	84.6 ± 27.1	3.6 ± 25.9	0.304
FEV1 (L/min)	1.9 ± 0.4	2.0 ± 0.9	0.1 ± 0.8	0.333
FEV1 (%) (predicted)	84.8 ± 18.3	85.5 ± 19.7	0.8 ± 11.7	0.619
FVC (L)	2.4 ± 0.5	2.4 ± 0.6	0.0 ± 0.4	0.866
FVC (%) (predicted)	84.6 ± 16.7	84.8 ± 19.1	0.2 ± 11.6	0.872
MIP (cmH2O)	69.5 ± 24.3	75.1 ± 24.2	5.6 ± 17.4	0.019
MEP (cmH2O)	111.3 ± 32.4	110.8 ± 25.8	− 0.4 ± 20.4	0.870
VT (mL) (at rest)	503.2 ± 156.0	487.4 ± 145.0	− 15.8 ± 139.0	0.399
VT (mL) (during exercise)	1,129.8 ± 277.2	1,211.3 ± 357.4	81.5 ± 265.9	0.026
RF (breaths/min) (at rest)	19.0 ± 5.3	18.5 ± 5.0	− 0.5 ± 5.2	0.489
RF (breaths/min) (during exercise)	35.8 ± 8.4	35.1 ± 7.5	− 0.7 ± 7.3	0.490
SpO2 (%) (at rest)	96.6 ± 1.7	97.0 ± 0.9	0.4 ± 1.5	0.057
SpO2 (%) (during exercise)	95.8 ± 3.0	95.8 ± 2.4	0.0 ± 1.7	0.874
VEQ	36.5 ± 5.1	35.1 ± 4.6	− 1.3 ± 4.9	0.048

*p* = comparison between pre- and post-PR

*Abbreviations*: *FEV1* Forced expiratory volume in 1 s, *FVC* Forced vital capacity, *MEP* Maximal expiratory pressure, *MIP* Maximal inspiratory pressure, *PR* Pulmonary rehabilitation, *RF* Respiratory frequency, *SpO2* Arterial oxygen saturation, *VEQ* Ventilatory equivalent, *VT* Tidal volume

### Effects of PR on cardiovascular parameters

The pre- and post-PR outcomes of the cardiovascular parameters are shown in Table 4. No significant differences were observed between pre- and post-PR measurements of the SVI (*p* = 0.144) and CI (*p* = 0.218) at rest. At peak exercise, the SVI and CI increased significantly from 52.1 ± 17.1 to 57.4 ± 15.4 mL/beat (mean difference: 5.3 ± 18.2 mL/beat; *p* = 0.034) and from 6.6 ± 2.6 to 7.4 ± 2.3 L/min/m^2^ (mean difference: 0.8 ± 2.6 L/min/m^2^; *p* = 0.025), respectively. Other CPET parameters also showed improvement, including O2P (from 8.4 ± 1.9 to 8.9 ± 2.1 mL/beat (mean difference: 0.5 ± 1.4 mL/beat; *p* = 0.017), WE (from 8.3 ± 1.5 to 8.8 ± 1.1 mL/min/W (mean difference: 0.5 ± 1.4 mL/min/W; *p* = 0.005), and VO2 at AT (from 672.4 ± 126.8 to 700.5 ± 134.2 mL/min (mean difference: 28.2 ± 95.6 mL/min; *p* = 0.032). During exercise, no significant differences were observed between pre- and post-PR measurements of HR (*p* = 0.180) and mean BP (*p* = 0.825).

**Table 4:** Effect of PR on cardiovascular responses to exercise

	**Pre-PR**	**Post-PR**	**Mean difference**	***p***
SVI (ml/min/m^2^) (at rest)	42.1 ± 11.2	42.0 ± 9.4	− 0.1 ± 10.8	0.144
SVI (ml/min/m^2^) (during exercise)	52.1 ± 17.1	57.4 ± 15.4	5.3 ± 18.2	0.034
CI (L/min/m^2^) (at rest)	3.4 ± 0.7	3.3 ± 0.7	− 0.1 ± 0.9	0.218
CI (L/min/m^2^) (during exercise)	6.6 ± 2.6	7.4 ± 2.3	0.8 ± 2.6	0.025
O2P (mL/beat)	8.4 ± 1.9	8.9 ± 2.1	0.5 ± 1.4	0.017
WE (mL/min/W)	8.3 ± 1.5	8.8 ± 1.1	0.5 ± 1.4	0.005
AT (mL/min)	672.4 ± 126.8	700.5 ± 134.2	28.2 ± 95.6	0.032
HR (beats/min) (during exercise)	127.2 ± 19.4	129.8 ± 19.2	2.6 ± 14.2	0.180
Mean BP (mmHg) (during exercise)	110.6 ± 13.7	111.1 ± 14.4	0.4 ± 14.8	0.825

*p* = comparison between pre- and post-PR

*Abbreviations*: *AT* Anaerobic threshold, BP Blood pressure, *CI* Cardiac index, *HR* Heart rate, *O2P* Oxygen pulse, *PR* Pulmonary rehabilitation, *SVI* Stroke volume index, *WE* Work efficiency

## Discussion

Our study showed significant improvements in patients with lung cancer who underwent PR. PR significantly improved exercise capacity, exertional symptoms, and HRQL. Although lung function parameters did not change significantly, there was a significant improvement in MIP, suggesting enhanced respiratory muscle function. Cardiovascular parameters, including the SVI, CI, WE, O2P, and AT, improved during exercise, highlighting the cardiovascular benefits of the PR program. These results suggest that an enhanced cardiopulmonary response to exercise improves exercise capacity and exertional symptoms, ultimately leading to reduced symptom burden.

The current study shows distinct demographic features of patients, including a predominance of early-stage lung cancer, a higher incidence in female and a low smoking rate. Lung cancer generally occurs more often in males than in females and is more prevalent among smokers. In Taiwan, the trend of lung cancer incidence might differ from global patterns due to various factors such as environmental influences, genetic predispositions, or lifestyle [17]. Chinese-food chefs have a 2.3-fold higher risk than non-chefs, and female not using fume extractors while cooking have a 3.5–12-fold higher lung cancer risk [17]. Recently, there has been a significant increase in lung cancer cases among Taiwanese female, particularly in early-stage lung adenocarcinoma. This trend might be linked to more nonsmokers undergoing low-dose computed tomography screening [17].

An important finding of this study was that PR resulted in improvements in MIP, VT, and VEQ during exercise. Respiratory muscles generate the pressure differences driving ventilation [18]. Respiratory muscle weakness can lead to poor ventilation efficiency, exercise capacity, and HRQL. Improved respiratory muscle strength is needed to increase VT [19]. In a previous study [19], we showed that PR improves respiratory muscle strength and VT, especially in patients with reduced respiratory muscle strength. Another notable finding was the improvement in VEQ following PR. VEQ represents the ability of the respiratory system to maintain a balance between ventilation and metabolism during exercise [14]. The improvement in VEQ suggests that PR enhances gas exchange efficiency and respiratory function during exercise. This may be attributed to improved cardiovascular fitness, better respiratory muscle coordination, and reduced respiratory effort, which contribute to more efficient ventilation and carbon dioxide removal [14]. The improvements in MIP, VT, and VEQ observed in this study provide evidence of the beneficial effects of PR on respiratory function in patients with lung cancer. These improvements likely contributed to improved exercise capacity and reduced exertional dyspnea following PR.

The observed improvement exclusively in MIP could be attributed to several factors. MIP is a direct measure of respiratory muscle strength, particularly the muscles involved in inspiration such as the diaphragm. The breathing exercises such as diaphragmatic breathing, are designed to strengthen these muscles [20]. Additionally, the measurement of MIP is sensitive and can detect even small changes in the strength of respiratory muscles [21]. MEP is the effort-dependent nature measurements can introduce bias, as maximal effort during measurements may be challenging to achieve [21]. Previous studies also showed that MIP but not MEP was associated with COPD severity [21]. Additionally, if participants had relatively well-preserved inspiratory muscle strength at baseline, the potential for improvement might be limited [19]. The effectiveness of PR in improving lung function parameters such as FEV1, FVC, and FEV1/FVC ratio is controversial [22, 23]. Previous studies have shown that PR improved these parameters in patients with poor lung function [23]. However, in our study, the patients' lung function were already above normal values, and we did not observe significant improvements in these parameters.

This study also showed that PR had a positive effect on cardiovascular parameters, including SVI, CI, WE, O2P and AT, during exercise. This suggests that PR not only improves respiratory function but also enhances cardiovascular performance. A previous study [24] reported that patients with cancer experience cardiac wasting, with structural and hemodynamic changes due to cancer-related cardiac wasting. Exercise training has been shown to improve cardiac function during exercise in patients with cancer [25]. A PR program incorporating exercise training and cardiovascular conditioning improves cardiac contractility and stroke volume, resulting in better oxygen delivery to tissues during exercise [12, 25].

O2P, WE, and AT not only serve as indicators of cardiac function, but also reflect the oxygen extraction capacity of peripheral muscles [12]. Exercise training is widely recognized for its ability to stimulate skeletal muscle growth, enhance mitochondrial function, and improve the oxygen extraction capacity of peripheral muscles [26]. Exercise training contributes to more efficient oxygen utilization in the peripheral muscles. Delayed anaerobic metabolism during exercise was observed after PR. In this study, we observed a significant reduction in leg soreness during exercise. Leg soreness is a common symptom experienced during physical activity that is often associated with muscle strain and fatigue. This suggests that exercise training can enhance muscular endurance and reduce discomfort during exercise.

Patients with lung cancer often experience cardiac or pulmonary comorbidities, which can diminish exercise performance, decrease physical activity levels, exacerbate muscle weakness, and increase symptoms [5]. A previous study demonstrated that an improvement in physical activity level was observed following PR [27]. Physical activity is also considered as an intervention for improving psychological well-being, anxiety and depression and maintaining the ability to perform daily activities [28]. Considering these significant benefits, physical activity is increasingly as a vital element of comprehensive cancer care [28].

Assessment of HRQL in lung cancer is important. Although there are several questionnaires about cancers in the past, many questionnaires are time-consuming and not easy to use. The European Organisation for Research and Treatment of Cancer Quality of Life Questionnaire-Core 30 (EORTC QLQ-C30) is commonly used in cancer research that it comprehensively encompasses physiological, respiratory, gastrointestinal, sleep functions, and financial concerns [29]. However, the EORTC QLQ-C30 is not specifically tailored for respiratory symptoms and is time-consuming, leading to its primary use in research rather than clinical practice. While the CAT was initially designed for COPD, it encompasses most respiratory symptoms, making it applicable to other pulmonary diseases such as pulmonary fibrosis [30] and coronavirus disease 2019 [31]. In our previous study, we showed that CAT also significantly reflected the changes in HRQL of lung cancer [4].

### Clinical implications

This study has important clinical implications as it demonstrates that PR can significantly improve cardiopulmonary function, exercise capacity, exertional symptoms, and HRQL in patients with lung cancer. Incorporating PR into the management of patients with lung cancer can improve overall physical well-being, activities of daily living, and HRQL.

### Study limitations

This study has several limitations. First, the sample size is relatively small. Its single-center design may have introduced selection bias. Multicenter studies with larger sample sizes are needed to confirm our findings. Second, this was a retrospective study. Prospective randomized controlled trials are needed to provide stronger evidence. Despite these limitations, we provide real-world evidence of the effectiveness of PR. Third, the relatively short 12-week follow-up period may not have been sufficient to determine the long-term effects of PR. Longer follow-up studies are needed to confirm the long-term effects of PR. Finally, all patients received PR in this study had non-small cell lung cancer (NSCLC), with no cases of small cell lung cancer (SCLC). Treatment strategies and prognoses significantly differ between SCLC and NSCLC. Therefore, the conclusions of this study are applicable exclusively to NSCLC and should not be generalized to SCLC.

## Conclusions

This study highlighted the benefits of PR in patients with lung cancer. PR effectively improved exercise capacity, exertional symptoms, and HRQL in these patients. Improved respiratory function (MIP, VT, and VEQ) and cardiovascular performance (SVI, CI, WE, and O2P) were also observed. Improvements in respiratory and cardiovascular function contribute to enhanced exercise performance, reduced dyspnea, and improved HRQL in patients with lung cancer. PR may be a valuable component in the management of patients with lung cancer.

## Data Availability

All data analysed during this study are included in this published article.

## Abbreviations

HRQL: Health-related quality of life
PR: Pulmonary rehabilitation
CAT: Chronic obstructive pulmonary disease assessment test
CPET: Cardiopulmonary exercise test
VO2: Oxygen uptake
VCO2: Carbon dioxide output
VT: Tidal volume
RF: Respiratory frequency
HR: Heart rate
BP: Blood pressure
WR: Work rate
VEQ: Ventilatory equivalent
MIP: Maximum inspiratory pressure
